# The Expression and Significance of Neuronal Iconic Proteins in Podocytes

**DOI:** 10.1371/journal.pone.0093999

**Published:** 2014-04-03

**Authors:** Yu Sun, Hongxia Zhang, Ruimin Hu, Jianyong Sun, Xing Mao, Zhonghua Zhao, Qi Chen, Zhigang Zhang

**Affiliations:** 1 Department of Pathology, Key Laboratory of Molecular Medicine, Chinese Ministry of Education, Shanghai Medical College, School of Basic Medical Science, Fudan University, Shanghai, P.R. China; 2 Department of Pathology, Weifang Medical University, Weifang, Shandong, P.R. China; 3 Institute of Health Sciences, Shanghai Institutes for Biological Sciences, Chinese Academy of Sciences, Shanghai, P.R. China; University of Leicester, United Kingdom

## Abstract

Growing evidence suggests that there are many common cell biological features shared by neurons and podocytes; however, the mechanism of podocyte foot process formation remains unclear. Comparing the mechanisms of process formation between two cell types should provide useful guidance from the progress of neuron research. Studies have shown that some mature proteins of podocytes, such as podocin, nephrin, and synaptopodin, were also expressed in neurons. In this study, using cell biological experiments and immunohistochemical techniques, we showed that some neuronal iconic molecules, such as Neuron-specific enolase, nestin and Neuron-specific nuclear protein, were also expressed in podocytes. We further inhibited the expression of Neuron-specific enolase, nestin, synaptopodin and Ubiquitin carboxy terminal hydrolase-1 by Small interfering RNA in cultured mouse podocytes and observed the significant morphological changes in treated podocytes. When podocytes were treated with Adriamycin, the protein expression of Neuron-specific enolase, nestin, synaptopodin and Ubiquitin carboxy terminal hydrolase-1 decreased over time. Meanwhile, the morphological changes in the podocytes were consistent with results of the Small interfering RNA treatment of these proteins. The data demonstrated that neuronal iconic proteins play important roles in maintaining and regulating the formation and function of podocyte processes.

## Introduction

Podocytes are one of the types of glomerular resident cells, which are characterized by their arborized cellular architecture, with thick major processes and thin foot processes. The thin foot processes and GBM cooperatively construct the glomerular filtration barrier, which can effectively prevent proteins from passing through. The fine cellular architecture of podocytes is often altered; when podocytes are injured in certain pathophysiological conditions or in nephropathies, which are called podocytopathies, such as process effacement, false microvillus structures that formed from excessive cytoplasmic spines extending and the fracture or lose of the foot processes. These alterations may increase the permeability of the glomerular filtration barrier and cause massive proteinuria. Therefore, the maintenance of the special morphology construction of the podocyte is thought to be essential for its normal function. This morphology not only forms the unique shape and filtration function but also plays important roles in material metabolism, cell movement, energy and intracellular information transmission [1]. The elucidation of the mechanism of podocyte foot process formation is important for determining approaches to the prevention and control of nephropathy.

The mechanism of podocyte foot process formation remains unclear. However, several current studies have shown that there are many common cell biological features shared by neurons and podocytes. Naoto Kobayashi et al. revealed that podocytes had an extraordinary similarity with neurons [2]. Both cells are highly differentiated with similar long and short cell processes that are equipped with highly organized cytoskeletal systems. Additionally, the two types of cells share the expression of structural and regulatory proteins, such as synaptopodin, drebin and desin [3]. Synaptopodin, which is the major component of the podocyte cytoskeleton, is primarily expressed in the site of the terminal processes of podocytes. Moreover, this protein can also be expressed in neuronal dendritic spines [4]. In addition, more recent data have shown that podocyte processes also share the expression of some special proteins, such as nephrin and podocin, with neuronal dendrites [5]–[8].

Moreover, some molecules that are abnormally expressed may be involved in the pathological states of brain and kidney tissues. For example, ubiquitin carboxy-terminal hydrolase L1 (UCH-L1), which is a member of the deubiquitination enzyme family, is specifically expressed in the brain, testis and kidney tissue under normal circumstances [9]–[12]. The abnormal expression of UCH-L1 in neurons is usually related to some degenerative diseases, such as Parkinson's disease, in which dopamine and other proteins are deposited in Lewy bodies, which resulted from a ubiquitination disorder [13]–[15]. In podocytes, our study and other laboratories have reported that UCH-L1 expression was elevated in diseased podocytes in several types of nephritis, which are related to the severity of nephropathy and proteinuria [16]. Enhancing UCH-L1 activity in membranous glomerulonephritis of rats could result in the inhibition of the ubiquitin-proteasome pathway, an increase in proteinuria and the progression of nephropathy [17]. Thus, the data may suggest the similarity of the pathogenesis between podocyte injuries and neuron lesions [18]. Among process-bearing cells, the neuron is the most intensely investigated so far. The careful comparison between neurons and podocytes may provide certain clues for understanding the pathophysiological mechanism of podocytopathy and nephropathy, as well as for providing novel therapeutic approaches.

The majority of the abovementioned studies were focused on podocyte differentiation proteins, such as nephrin, podocin and synaptopodin. To investigate process formation in greater detail, we hypothesized that some of the neuronal iconic molecules are also expressed in podocytes and that these molecules could play an important role in podocyte differentiation. In this study, we attempted to determine whether some neuronal iconic molecules, such as NSE (Neuron-specific enolase), nestin, NeuN (Neuron-specific nuclear protein) and S100, were expressed in podocytes through cell biology and immunohistochemistry techniques. The results confirmed that these neuronal iconic proteins were expressed in podocytes and played an important role in the formation of foot processes. These data indicated that these neuronal iconic proteins could be a critical regulator for podocyte function and a novel target in therapeutic approaches for podocytopathy and nephropathy.

## Materials and Methods

### Ethics statement

Permission to use the tissue sections for research purposes was obtained and approved by the Ethics Committee from Shanghai Medical College, Fudan University, China, and a written consent form was obtained from all patients. The animal number and the protocols of the animal experiments were approved by Experimental Animal Ethics Committee of Shanghai Medical College, Fudan University, China.

### Antibodies

Antibodies were purchased as follows: **Primary antibodies:** rabbit-anti-UCH-L1 (AB1761,MILLIPORE), rabbit-anti-synaptopodin (S9442, Sigma-Aldrich), mouse-anti-α-actinin4 (MAB1682, MILLIPORE), mouse-anti-nestin (MAB2763, R&D), rabbit-anti-NeuN (ab104225, Abcam), mouse-anti-S100 (ab14849, Abcam), rabbit-anti-NSE (ab53025, Abcam), mouse-anti-nephrin (sc-166574, Santa Cruz Biotechnology), mouse-anti-WT-1 (ab96792, Abcam). **Secondary antibodies:** peroxidase-conjugated goat-anti-mouse IgG, peroxidase-conjugated goat-anti-rabbit IgG, fluorescein (FITC)-conjugated goat-anti-rabbit/mouse IgG, cy3-conjugated goat-anti-mouse/rabbit IgG all from Protein Tech Group Inc., Chicago, USA, HRP-goat-anti-mouse IgG, HRP-goat-anti-rabbit IgG from Long Island Biotech Co., LTD, Shanghai, China.

### Immunohistochemistry

Two cases of kidney tissue from renal needle biopsies with acute tubulointerstitial lesions (the glomeruli structures were almost normal) were collected from the nephrosis laboratory, and one case of brain tissue was obtained from the autopsy of a volunteer cadaver donation with colon cancer, Department of Pathology, Shanghai Medical College, School of Basic Medical Science, Fudan University, in accordance with local ethical guidelines. Four-micrometer thick paraffin sections were deparaffinized, endogenous peroxidase was removed by 0.3% H_2_O_2_-methanol and antigen retrieval was performed by microwave boiling (10 mM citrate buffer, pH 6.1). Unspecific binding was blocked (10% normal goat serum, 30 minutes, 37°C). Then, the sections were probed with primary antibodies against NSE (1∶50), UCH-L1 (1∶200), NeuN (1∶50), nestin (1∶50), S100 (1∶100), nephrin (1∶100), synaptopodin (1∶200) and α-actinin-4 (1∶100) for 1 hour at 37°C and then incubated overnight at 4°C, which was followed by incubation with biotinylated secondary antibodies (1 hour, 37°C). DAB (Sigma) was used as the chromogen.

### Podocyte Culture

A conditionally immortalized mouse podocyte line (MPC5) (a gift from Dr Xu, Children's Hospital of Fudan University [19]) was cultured under permissive conditions (33°C, 5% CO_2_, RPMI 1640, 10% FCS, 50 U/ml IFN-γ (Sigma)) for 5–8 days while podocytes were in an undifferentiated state. Later, podocytes were cultured for differentiation under non-permissive conditions (37°C, 5% CO_2_, RPMI 1640, 10% FCS, without IFN-γ) on coated collagen I (Sigma) cell ware, and 10–14 days later, we observed podocytes with long spindle shapes and a small amount of branches under a microscope (Nikon, Japan), which was consistent with the podocyte morphology characteristics that were reported in the literature.

### Primary cortical neuron culture

Primary cortical neurons were prepared from embryonic *Sprague-Dawley* rats. Fetal rat cerebral tissue was isolated, and the cortex was removed into a glass Petri dish with pre-cooled D-Hank's liquid. The cortex was cut into 1-mm^3^ blocks and placed into a sterile centrifuge tube. After 1000 RPM instantaneous centrifugation, the supernatant was collected, 0.125% trypsin-1 mM EDTA was added to a total volume of 5 ml, and the sample was digested at 37°C for 20 minutes. Then, the digestion was suspended by adding a corresponding volume of DMEM containing 10% FCS. The sample was then centrifuged for 5 min at 1000 RPM, the supernatant was discarded, and 5 ml of DMEM was added. The cell suspension was then pipetted with a capillary straw repeatedly until no blocks could be observed, and the liquid was in a turbid state. The cell suspension was finally filtered with a 200mesh filter. Neurons were seeded at 5–6×10^5^ cells per well and cultured in Neurobasal medium (Gibco, USA) in a humidified incubator at 37°C and 5% CO_2_.

### Western Blotting

Podocytes were lysed quickly using 100 μl 2×SDS buffer, boiled for 5 minutes and then stored at −20°C until use. Portions (15 μl) of the supernatant, including soluble cell proteins, were subjected to 10% SDS–PAGE and then transferred to PVDF membranes (Millipore). The membranes were blocked using 5% BSA (Shanghai HaoZe Biotech Co., Ltd, China) before incubation with primary antibodies, which were diluted in blocking liquid. The membranes were probed with antibodies against UCH-L1 (1∶2000), synaptopodin (1∶2000), α-actinin4 (1∶1000), nestin (1∶1000), NeuN (1∶500), S100 (1∶500), NSE (1∶500) and GAPDH (1∶1000) as a loading control for 1 hour at room temperature. Following binding, the proteins were detected with peroxidase-conjugated secondary antibodies. The immunoreaction was visualized by ECL SuperSignal (Thermo) and by film exposure.

### Double immunofluorescence labeling

Podocytes were seeded on sterile cover slips and cultured at 37°C in 95% air and 5% CO_2_ until the cell density reached 50%–60%. Pre-cooled acetone was used as the stationary fixing liquid, and unspecific staining was blocked by 2.5% BSA for 30 minutes at room temperature. Primary antibodies, including NSE (1∶50), UCH-L1 (1∶100), NeuN (1∶50), nestin (1∶50), S100 (1∶50), synaptopodin (1∶100) and α-actinin-4 (1∶50), were incubated for 1 h at 37°C, followed by incubation with FITC-conjugated secondary antibodies, as well as WT1 (1∶100, as the nuclear marker of podocytes) with Cy-3-conjugated secondary antibodies for 45 minutes at 37°C. Cover slips were examined using a fluorescence microscope (Nikon, Japan), and merged images were formed using the Adobe Photoshop CS5 software.

### Immunofluorescence staining of F-actin

Podocytes were seeded on sterile cover slips and cultured at 37°C in 95% air and 5% CO_2_ until the cell density reached 50%–60%. Next, 4% polyformaldehyde–PBS was used as the stationary liquid for 10 minutes, and the cells were then treated by 0.1% Triton X-100-PBS for 5 minutes. Unspecific staining was blocked by 3% BSA for 20 minutes at room temperature. Fluorescent phallotoxins (F-actin) (1.5 IU/200 μl, Invitrogen) were added and incubated at 37°C for 30 minutes. The cover slips were then examined with a fluorescence microscope (Nikon, Japan).

### RNA interference

Shortly before transfection, podocytes were seeded 1.5–6×10^5^ (37°C cultured) per well in 2300 μl 1640 RPMI, which contained serum. Podocytes were incubated under normal growth conditions (37°C, 5% CO_2_). In total, 150 ng siRNA (Qiagen) was diluted in 100 μl 1640 RPMI without serum. Then, 12 μl HiPerFect Reagent (Qiagen) was added and mixed by vortexing. Samples were incubated for 5–10 minutes at room temperature for complex formation. Complexes were added drop-wise onto podocytes, and the plate was gently swirled. These podocytes were then incubated under normal growth conditions (37°C, 5% CO_2_), and gene silencing was monitored after 72 h. The medium was changed after 6 h.

### Statistical Analysis

Statistical analyses were performed using the GraphPad Prism 5 software. Statistical comparisons between two data points were made using Student's t-test and between three or more data points using ANOVA combined with post-hoc Bonferroni's multiple comparison tests. The data were expressed as the mean ± SD. When p<0.001, the result was considered statistically significant. All experiments were performed in triplicate.

## Results

### Location of neuronal iconic proteins expression in human renal tissues and human brain tissues

**Figure 1:**
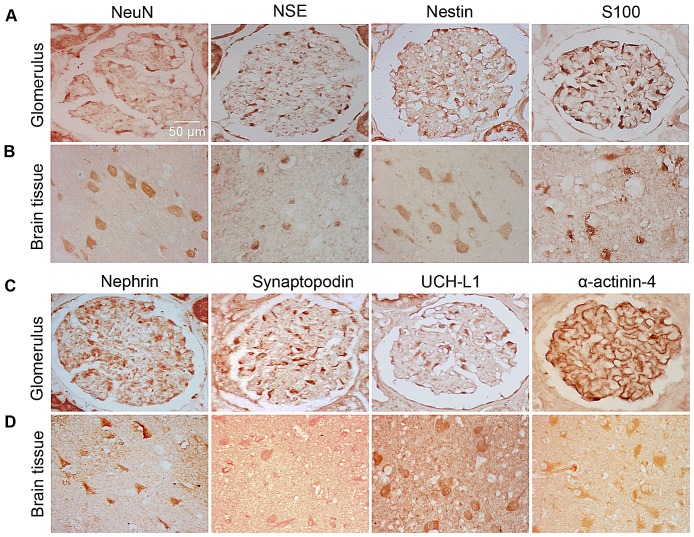
Location of neuronal iconic protein expression in human renal tissues and in human brain tissues. Immunohistochemical staining of NeuN, NSE, nestin, S100, nephrin, synaptopodin, UCH-L1 and α-actinin-4 were positive in the glomerulus, with positive cells primarily localized around the glomerular capillary loop, approximately at the site of podocyte distribution (A, C), as well as in the polymorphic neurons in brain tissues (B, D). Elivison×400.

Using immunohistochemistry, we detected the distribution of NSE, NeuN, nestin, S100, nephrin, UCH-L1, synaptopodin and α-actinin-4 in normal glomeruli compared with human normal brain tissues as the control. As displayed in Fig. 1, the results indicate that all of the abovementioned indicators were positive in brain neurons. These indicators were also positively expressed in the podocytes of glomeruli. The positive cells were primarily in the outer aspect of the glomerular capillary loop, which is the site of podocyte distribution. Meanwhile, these cells also proved to be podocytes with the positive expression of nephrin and synaptopodin (usually recognized as podocyte markers). In contrast, the cells in mesangial areas were almost all negative (Fig. 1A, C). These neuronal iconic proteins are expressed in glomerular podocytes.

### Neuronal iconic proteins expressed in mouse podocyte and neurons

**Figure 2:**
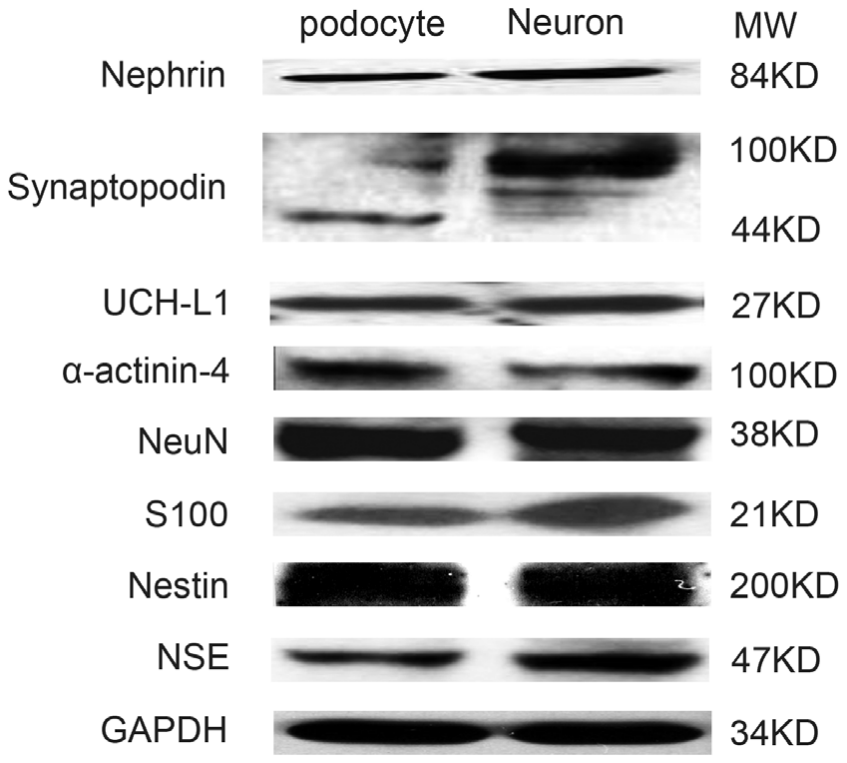
Neuronal iconic proteins that are expressed in mouse podocytes and brain neurons. Neuronal iconic proteins NSE (47 kDa), NeuN (38 kDa), nestin (200 kDa), S100 (21 kDa), nephrin (84 kDa), synaptopodin (44 kDa in podocyte, 100 kDa in neuron), UCH-L1 (27 kDa) and α-actinin-4 (100 kDa) were detected by Western blotting in both podocytes and brain neurons. The data are presented from at least three individual experiments that were performed in duplicate.

We extracted total proteins from cultured mouse podocytes and rat neurons. Western blotting detected the same proteins in neurons and podocytes as abovementioned. The results indicated that all of these proteins were expressed in both podocytes and brain neurons (Fig. 2). The molecular weights of NSE, NeuN, nestin and S100, as well as nephrin, UCH-L1 and α-actinin-4, were of the same size in both podocytes and neurons, whereas synaptopodin is 44 kDa in podocytes and 100 kDa in neurons, which is consistent with Dr. Mundel's report [7].

### Immunofluorescence detection of neuronal iconic proteins in cultured mouse podocytes

**Figure 3:**
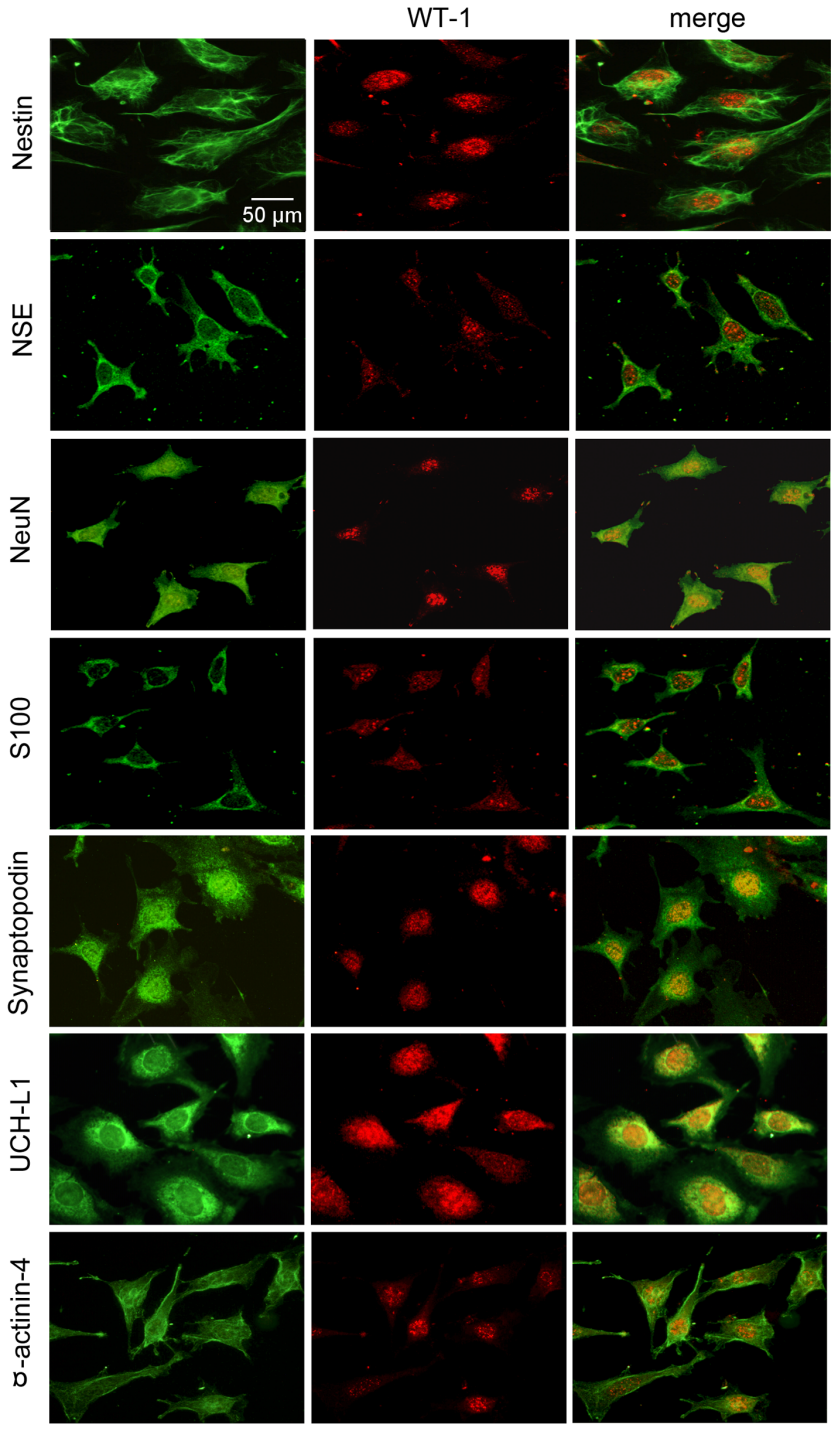
Immunofluorescence detected neuronal iconic proteins in cultured mouse podocytes. In the slides of cultured podocytes, nestin, NSE, NeuN, S100, synaptopodin, UCH-L1 and α-actinin-4 are positively expressed (green fluorescence), with WT-1 as a specific podocyte marker (red fluorescence); the merged images demonstrate the overlap of green and red fluorescence (yellow). IF×400.

Using double labeling of neuronal iconic proteins (green fluorescence) and WT-1 as podocyte marker (red fluorescence), the confocal merged images indicated that the neuronal iconic proteins NeuN, NSE, nestin, UCH-L1, S100 and α-actinin-4 were positively expressed in cultured mouse podocytes *in vitro* (Fig. 3). In addition, other podocyte marker proteins, such as nephrin and synaptopodin, were positive in podocytes.

### The effects of RNA interference of neural iconic proteins on podocyte morphology

**Figure 4:**
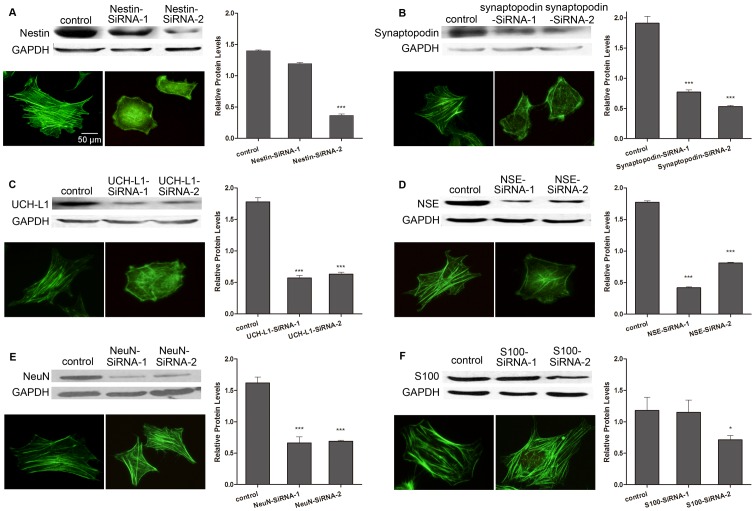
The effects of RNA interference of neuronal iconic proteins on podocyte morphology. Following RNA interference of nestin (A), synaptopodin (B), UCH-L1(C) and NSE (D) separately for 72 hours, Western blotting showed a decrease in the protein level of these indicators in treated podocyte compared with the control cells. Additionally, the statistical results show significant differences between the treated groups and the control group; the F-actin staining in treated podocytes showed significant morphological changes and F-actin misalignment compared with the control cells (A, B, C, D). The inhibition of NeuN (E) and S100 (F) mRNA resulted in few differences in the morphology and F-actin arrangement (E, F) compared with the control cells (compared with. Control *p<0.05, ***p<0.001). The data are presented from at least three individual experiments that were performed in duplicate. Fluorescence Phallotoxins ×400.

To clarify the role that these neuronal iconic proteins play in process formation in podocytes, we further used RNA interference of neuronal iconic proteins by siRNA in podocytes for 72 hours separately, including NSE, nestin, NeuN, S100, synaptopodin and UCH-L1, and observed the morphologic changes in podocytes. The expression level of the abovementioned indicators decreased significantly in separately treated cells (Fig. 4). Then, we stained the cytoskeleton with F-actin in both control and treated podocytes. The control podocytes displayed a polygonal structure, and the actin fibers were orderly arranged in radial and fasciculate patterns in the cytoplasm. By contrast, the morphology of podocytes significantly changed when cells were treated with siRNA with targeted RNA interference of NSE, nestin, synaptopodin and UCH-L1. The polygonal shape of cells became obtuse, the thin cytoplasmic processes clearly decreased and the intracellular actin fibers became misaligned (Fig. 4A, B, C, D). The results indicated that nestin, NSE, synaptopodin and UCH-L1 played important roles in maintaining the formation of processes in podocytes. However, when NeuN and S100 mRNAs were inhibited, the polygonal morphology of podocytes was not changed, and the F-actin arrangement remained organized (Fig. 4E, F), which suggested that NeuN and S100 proteins might have other biological functions than process formation in podocytes.

### The morphological changes of podocytes that were treated with Adriamycin

**Figure 5:**
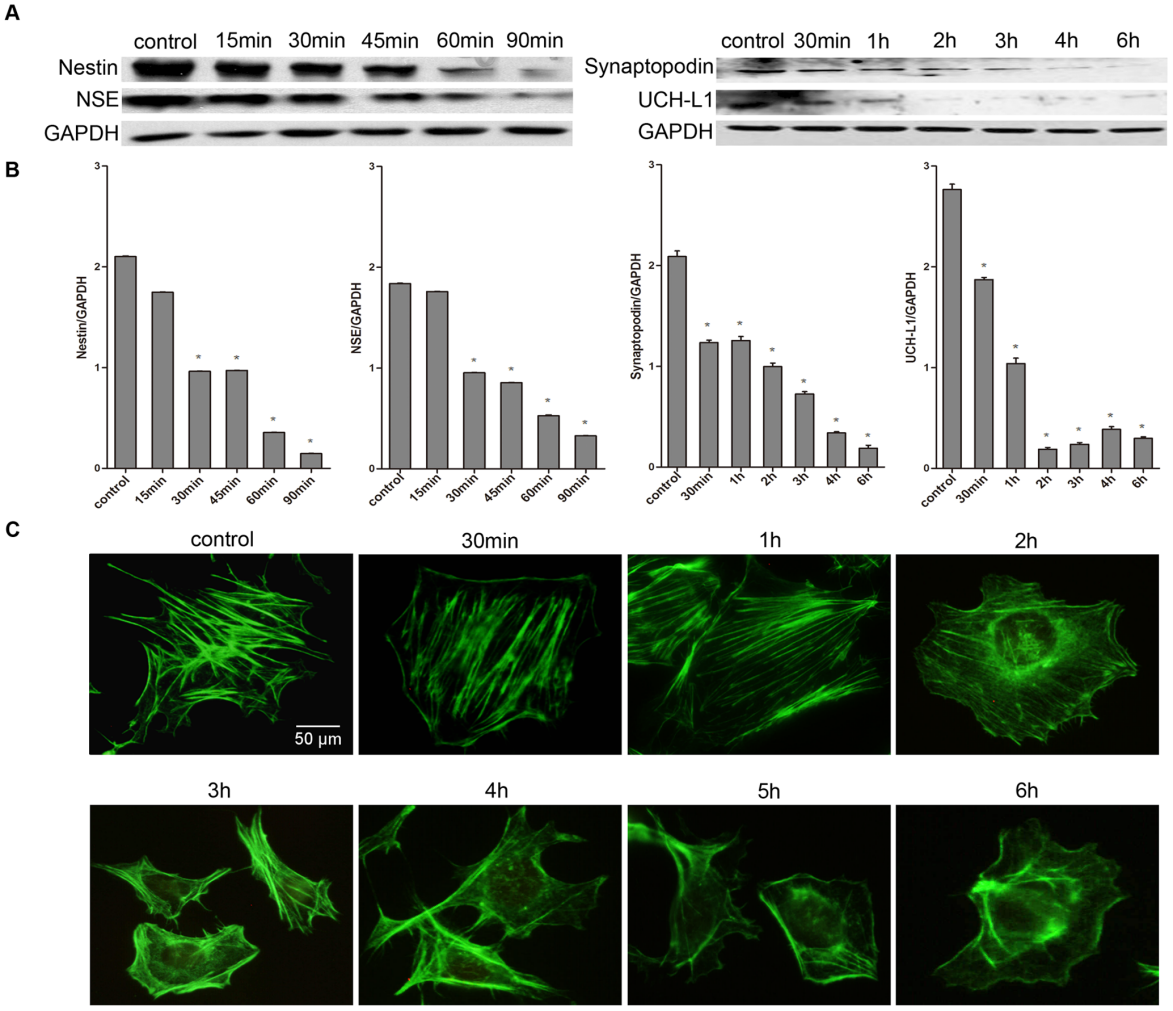
The morphologic changes of podocytes that were treated with Adriamycin. The podocytes were treated with Adriamycin (20 μg/ml) for different times. The expression levels of nestin, NSE synaptopodin and UCH-L1 continued to decrease in correlation with the stimulation time (A). The statistical results show significant differences between the controlled and treated groups (B) (compared with. control, *p<0.001). Two hours after treating the cultured mouse podocytes with Adriamycin (20 μg/ml), the cytoskeletons changed significantly, and as the stimulus time increased, the destruction of the cytoskeleton and morphology became more severe (C). The data are presented from at least three individual experiments that were performed in duplicate. Fluorescence Phallotoxins ×400.

To further investigate the changes of these neuronal iconic proteins in injured podocytes, we treated the cultured mouse podocytes with Adriamycin (20 μg/ml) for various periods. The results showed that the podocyte morphology did not change significantly when stimulated for 30 min and 1 h; however, the podocytes became swollen as actin fibers became disordered when the stimulation lasted for 2 hours. When the stimulus lasted more than 2 hours, the disordered arrangement of the cytoskeleton became more severe (Fig. 5C). Meanwhile, the Western blotting showed that the decrease in the protein expression of nestin, NSE, synaptopodin and UCH-L1 was dependent on the time of stimulation (Fig. 5A). These results indicate that when podocytes were damaged, the expression of these neuronal iconic proteins were also affected, which resulted in disordered cytoskeleton, changes in cell morphology, clinical proteinuria and other symptoms. These data further proved that neuronal iconic proteins might play an important role in maintaining the special morphology and functions of podocytes, as well as in the pathogenesis of podocyte injury.

## Discussion

Mesangial, endothelial and glomerulus visceral epithelial cells (podocytes) are three inherent cells of glomeruli. Damage to any one of these three cell types will cause kidney diseases. When mesangial or endothelial cells are damaged, the surrounding normal cells will begin the compensatory repair response with proliferation and hyperplasia. However, because of the highly differentiated characteristics and limited proliferative capability of podocytes, which are similar to neurons, podocytes cannot effectively recover from damage. Therefore, podocyte injury becomes a pivotal factor during the progression of glomerular disease [20], [21]. A study demonstrated that when 20%–40% podocytes were damaged, mild proteinuria would happen; when more than 60% of the podocytes were damaged, there was severe proteinuria and glomerular sclerosis [22]. All of the damaged podocytes have morphological changes as cell bodies swell, inclusive vacuoles increase and the foot processes fuse. Shirato once indicated that the molecular rearrangement of the cytoskeleton of podocyte foot processes was a key factor in the morphologic changes of the foot processes during podocyte injury [23]. Therefore, determining the formation and regulation mechanism of the podocyte cytoskeleton is essential for podocyte injury research and for proteinuria and glomerular sclerosis treatments.

In podocyte injury research, certain specific proteins, such as nephrin, podocin, UCH-L1 and synaptopodin, have been found to be involved in the formation of foot processes. These proteins are often ectopic expressed in a variety of nephropathies. Nephrin is a podocyte biomarker [24]. Nephrin was significantly reduced in the early stage of rat diabetic nephropathy. Podocytes also exhibited mild swelling, foot process broadening and effacement, which were associated with significant proteinuria [25]–[27]. Synaptopodin was originally found associated with actin filaments in podocyte foot processes [7], [28] and is thought to be vitally important for the morphology and function of podocyte processes. Therefore, synaptopodin is also considered a mature phenotype marker of podocytes [29]. In glomerular lesions, synaptopodin expression decreased from normal, MCD, to IgAN, MsPGN and to FSGS in turn. This observation suggested that the change in synaptopodin influenced the structure and function of podocytes, which play an important role during the progressive course of glomerular diseases [30], [31]. Podocin is localized in the slit diaphragm and is another molecular marker of mature podocytes. Podocin binds with nephrin and Neph1 to form a special signaling complex in foot processes, which is necessary for the structure and function of podocytes [32]. The reduction of podocin expression is also a symbol of podocyte injury [33].

These proteins also have been reported to be highly specifically expressed in neurons, particularly in the dendritic synapses and not in the axon [3]. These proteins play an important role in neuronal differentiation and synapse function. These proteins are also involved in neural lesions [10], [15], [18], [34], [35]. Naoto Kobayashi et al. indicated that [3], in addition to the co-expression of certain common proteins, these two cells shared other identical biological characteristics, such as 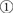 Both cells develop microtubule-based thick processes with branching morphology and have thin actin-based projections. 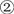 Both cells have the same mechanism of process formation, which is positively regulated by phosphorylation of microtubule-associated proteins. 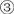 Both cells share the machinery for intracellular trafficking of membranous vesicles and cytoskeletal elements [3], [23]. There is currently growing evidence from our work and other laboratories that supports this concept that podocytes and neurons share the same regulatory mechanism for cell growth and development.

The proteins abovementioned, including nephrin, podocin and synaptopodin, are considered special podocyte proteins, which can also be expressed in neurons. Furthermore, as we speculated, there are many neuron-specific proteins, such as NSE, NeuN, nestin and UCH-L1, S100, that should also be expressed in podocytes. However, such reports are rarely seen. In the present study, we discovered that some neuronal iconic proteins, including NSE, nestin, NeuN, UCH-L1 and S100, could be found in human kidney tissue and in cultured podocytes, which indicated that neuronal specific proteins are expressed in podocytes.

Neuronal iconic proteins play an important role in neuron development, particularly in the formation of synaptic structures, as well as in the pathogenesis of neural diseases. NSE is a glycolytic enzyme that is primarily localized to the neuronal cytoplasm and synaptic plasma membrane [36], [37]. NSE was reported to be associated with the cytoskeleton and to play important roles in cell growth, proliferation and survival in neurons [38]. Immunocytochemical studies demonstrated that NSE was associated with microtubules in normal rat and mouse brains [39] and in some tumor cells [40]. Nestin, which belongs to the class IV intermediate filament proteins, is distributed in the cytoplasm and is involved in the construction of the cytoskeletal structure. Nestin is primarily expressed in undifferentiated neuronal cells, which have the ability for cell division, such as nerve stem cells. Nestin is a marker for early primitive nerve cells [41]. Nestin re-expression in ischemic brain tissue may enhance anti-ischemic therapy and increase cell survival and cytoskeleton stabilization in neurons [42]–[46]. UCH-L1 is a deubiquitination enzyme. UCH-L1 is primarily expressed in brain nerve cells and is recognized as a neuronal iconic protein. Recently, studies found that UCH-L1 is an important regulator of synaptic structure [47]. The inhibition of UCH-L1 could produce significant and dramatic alterations in synaptic protein distribution and spine morphology, which increased spine size and decreased spine density. UCH-L1 was found to be downregulated in early Alzheimer's brains. UCH-L1 overexpression in the mouse Alzheimer's model could help to recover synaptic function and improve cognitive function of mice [15]. Synaptopodin, as abovementioned, has also been confirmed to be necessary for synaptic structure in the brain [4], [7]. Using immunohistochemical methods, synaptopodin was found distributed in the postsynaptic densities and in the associated dendritic spines in exclusively telencephalic synapses; this distribution of synaptopodin is fully consistent with the distribution of actin [7]. The change in synaptopodin expression is also closely associated with neuropathy [48].

As discussed above, from the viewpoint of both cells sharing same mechanism of process formation, we supposed that the expression of these proteins in podocytes should closely associate with the regulation of morphology and function of foot processes. Therefore, we knockdown the expression of NSE, nestin, UCH-L1 and synaptopodin. The results showed that there appeared significant changes in podocytes, with blunt cell edges, decreased dendritic processes and a disordered arrangement of intracellular actin fibers. This observation suggests that NSE, nestin, UCH-L1 and synaptopodin play an important role in maintaining the special shape of podocytes dendritic processes. In fact, all of these proteins are altered in kidney diseases [20], [30], [49]. For example, in our previous work and in other laboratories, UCH-L1 expression was upregulated in several types of nephritis in podocytes [16], [20]. In this study, we blocked UCH-L1 in cultured podocytes; the changes in these cells paralleled the observations in human nephritis. These data strongly support the hypothesis that these neuronal iconic proteins also play an important role in podocyte injury. These proteins are a potential novel target for studies of podocyte injury and podocytopathy.

In addition, the expression of NeuN and S100 were also downregulated in podocytes; however, there were no significant changes in the cell cytoskeleton. This observation prompted the thought that not all of the neuronal proteins participated in cytoskeleton construction. Some of these neuronal proteins might have other biological functions in podocytes.

After treatment with Adriamycin, severe damage of the podocyte cytoskeleton was observed, which notably resembled the morphological changes in cultured podocytes with the blocked expression of NSE, nestin, UCH-L1 and synaptopodin. Simultaneously, the Western blotting showed that the protein levels of NSE, nestin, UCH-L1 and synaptopodin decreased in a time-dependent manner. This decrease may be the responsive reaction of podocytes because of the destruction of the cytoskeleton and the reduction of some associated proteins. These studies further proved that NSE, nestin, UCH-L1 and synaptopodin are important regulators that are involved in the mechanism of process formation. However, the molecular mechanisms of the process still require further investigation.

In summary, in the present work, some neuronal iconic proteins, such as NSE, nestin, NeuN and UCH-L1, could be expressed in podocytes. The reduction of these proteins expression by mRNA block or by stimulation of Adriamycin could induce the impairment of podocytes cytoskeleton and its morphology. This data proves that neuronal iconic proteins are important for the morphology and function of podocytes. In recent years, there has been great progress in the mechanism of nerve injury. Therefore, we can learn from the research progress of the nervous system, which is instructive for us to understand and clarify the podocyte injury mechanism. This research will be a good foundation for the further study of the pathogenesis of podocyte injury and for the exploration of novel therapeutic targets for nephropathy.
